# Identification and validation of a twelve immune infiltration-related lncRNA prognostic signature for bladder cancer

**DOI:** 10.18632/aging.203889

**Published:** 2022-02-14

**Authors:** Chen-Qian Liu, Qi-Dong Xia, Jian-Xuan Sun, Jin-Zhou Xu, Jun-Lin Lu, Zheng Liu, Jia Hu, Shao-Gang Wang

**Affiliations:** 1 Department and Institute of Urology, Tongji Hospital, Tongji Medical College, Huazhong University of Science and Technology, Wuhan 430030, China

**Keywords:** bladder cancer, TCGA, lncRNA, immune infiltration, prognostic signature

## Abstract

The prognosis of bladder cancer patients is strongly related to both the immune-infiltrating cells and the expression of lncRNAs. In this study, we analyzed the infiltration of immune cells in 403 bladder cancer samples obtained from TCGA by applying the ssGSEA to these samples, then dividing them into high/low immune cell infiltration groups. Based on these groupings, we found 404 differentially expressed immune infiltration-related lncRNAs, which were successively analyzed by univariate Cox regression, then Least Absolute Shrinkage and Selection Operator (LASSO), and finally stepwise multiple Cox regression. Then 12 differentially expressed immune infiltration-related lncRNAs were identified and used to construct a prognostic signature for bladder cancer. Subsequently, Kaplan-Meier analysis, univariate Cox regression, multivariate Cox regression, and multivariate time-dependent ROC analyses (for 1, 3, 5 years) all revealed that this signature performed well in predicting overall survival and served as an independent prognostic factor for patients with bladder cancer. Finally, both TIMER and CIBESORT showed that this 12-lncRNA prognostic signature for bladder cancer was associated with the infiltration of immune cell subtypes. Besides, nomogram considered risk score and clinical characteristics was assembled and showed great performance. More importantly, we found our signature could well distinguish the drug response of patients with bladder cancer. High risk patients showed a better response to cisplatin, doxorubicin, and anti- CTLA4 immunotherapy, low risk patients showed a better response to methotrexate and anti-PD1 immunotherapy compared with each other.

## INTRODUCTION

Bladder cancer (BC), a forth common cancer in men, counts on high incidence and mortality rate, estimated 549,000 new cases and 200,000 deaths in 2018, globally [1, 2]. Under the disease management of intravesical BCG instillations for NMIBC and radical cystectomy or chemotherapy for MIBC, or further several novel therapies such as immunotherapy, oncolytic viruses, it has been reported that the survival rate of bladder cancer within 5 years at all stages is no more than 20% [3]. Under the current diagnosis and treatment of bladder cancer, it is urgent to identify more reliable diagnosis and prognostic indicators.

Recent years, we’ve gradually come to know that the tumor microenvironment (TME) plays an essential role in tumor differentiation, tumor epigenetics, immune evasion, and even treatment resistance [4]. The state of host innate immune system and the proportion of local infiltration of different types of immune cells are critical factors of the TME [5]. Notably, the tumor immune micro-environment is similar with the immune infiltration of chronic inflammation, containing multiple different types of immune cells [6]. Immune infiltration in TME have been proved to have significant influence on both tumor-promoting and suppressing activities. Other studies also suggest that the density of immune cells is associated with the immune evasion and treatment resistance of breast cancer [7]. Among of these immune cells, lymphocytes are the main type of inflammatory immune cells in TME [8]. Besides, the infiltration of CD8+ T and CD4+ T cells is also reported with a significant influence on the prognosis of bladder carcinoma [9, 10]. Specifically, tumor infiltrating CD8+ T cells have anti-tumor function and show a positive effect on prognosis of many tumors [11–13]. Also, tumor associated macrophages play an essential role in bladder cancer, especially M2 macrophages [14]. As for dendritic cells (DC) and other antigen-presenting cells (APC), they play a significant role in the biological process of tumor antigen presentation [15, 16].

Long non-coding RNA (LncRNA) is a class of non-coding RNAs with transcripts longer than 200 nt, which do not translate proteins but regulate gene expression by multiple mechanisms in the form of RNA [17]. Meanwhile, lncRNAs play significant role in the progression and prognosis in human diseases, especially in cancer [18]. For example, in a recent meta-analysis, Quan, et al. reported that lncRNAs were associated with the prognosis of patients with bladder cancer and could perform as an effective prediction factor for the overall survival of BC patients [19]. Among these lncRNAs, UCA1 was considered to serve as an efficient biomarker in the diagnosis of bladder cancer, while the abnormal expression of HOTAIR and GAS5 was associated with a poor prognosis including elapse-free survival (RFS), disease-free survival (DFS), r and disease-specific survival (DSS) [19]. Furthermore, lncRNAs were reported to regulate the immune infiltration directly or indirectly [20]. For instance, it has been reported LNC-INSR could enhance Treg cells differentiation and promote immunosuppression in childhood acute lymphoblastic leukemia [21]. Recent studies have also shown that lncRNAs have a high frequency and cell-type-specific presence in different type of immune cells, and the expression pattern of key lncRNAs is also determined to be related to immune infiltration in TME [22].

Therefore, we aimed to screen immune-infiltration-related lncRNAs in BC patients. In this research, we comprehensively assess the immune infiltration of BC patients by two methods including single sample gene set enrichment analysis (ssGSEA) and ESTIMATE algorithm. Then identified differential immune subtype BC patients and discovered differentially expressed immune infiltration related lncRNAs. Finally, we developed a 12 differentially expressed immune-infiltrating related lncRNAs signature and demonstrated the correlation between risk score calculated by the signature and tumor microenvironment in bladder cancer.

## RESULTS

### Identification of bladder cancer immune subgroups

**Figure 1:**
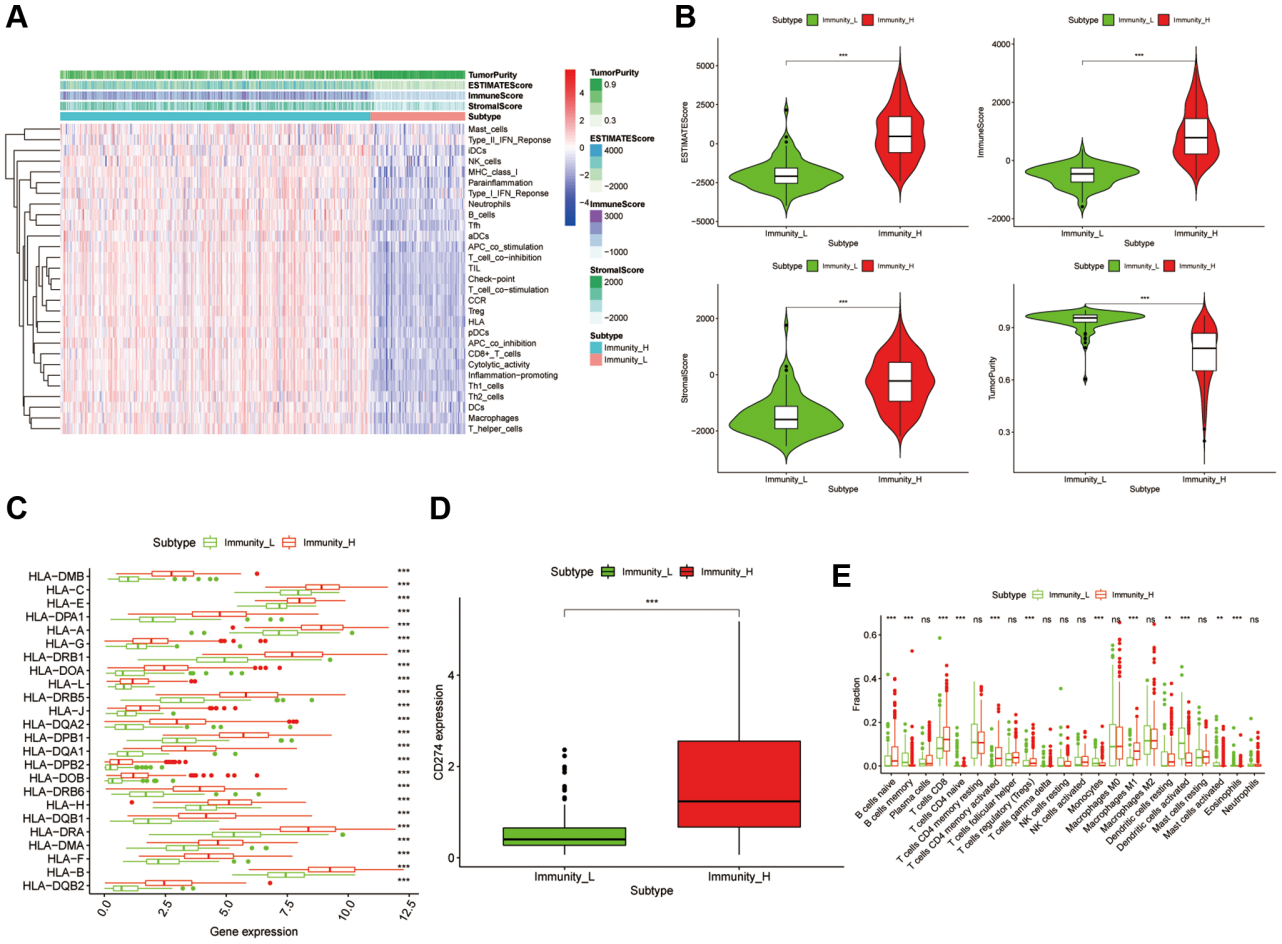
**Construction and verification of bladder cancer clustering by immune infiltration.** (**A**) The immune cells were highly infiltrated in the cluster1 group, which was named as the Immunity_H (high immune cell infiltration group), and the low expression one in the cluster2 group was named as the Immunity_L (low immune cell infiltration group). The Tumor Purity, ESTIMATE Score, Immune Score and Stromal Score of each sample gene were also displayed with the grouping information by using ESTIMATE's algorithm. (**B**) The box-plot showed a statistical difference in Tumor Purity, ESTIMATE Score, Immune Score and Stromal Score between the two groups (*p* < .01). (**C** and **D**) In Immunity_H (red), the expression of HLA family genes and CD274 were all significantly higher than that in the Immunity_L (green) (*p* < 0.001). (**E**) The statistical chart showed the proportion difference of each immune cell between the Immunity_H (red) and the Immunity_L (green), after using the CIBERSORT method.

A total of 405 bladder cancer patients from the TCGA-BLCA with 430 transcriptome profiles were obtained (Normal = 19, Tumor = 411). The transcriptome profiles of these samples were conducting ssGSEA analysis to evaluate the immune infiltration and immune related functions. Notably, there were 29 immunological marker gene sets, including immunological cell subtypes, pathways or functions associated with immune, have been used to perform the ssGSEA analysis. Then the samples were clustered into two groups according to the immune infiltration results, named accordingly, high immune cell infiltration cluster (Immunity H, *n* = 315) and low immune cell infiltration cluster (Immunity L, *n* = 96) (Figure 1A). The box chart also showed a significant higher Immune Score, Stromal Score, and ESTIMATE score in Immunity H group, while the Immunity L group showed a higher Tumor Purity (Figure 1B). Meanwhile, the expression values of CD274 (PD-L1) and HLA family was discovered significantly higher in Immunity H group than that in Immunity L group (Figure 1C and 1D, *p* < 0.001). Moreover, the CIBERSORT algorithm was used to verify the reliability of the unsupervised immune subgroups and found that there were higher infiltration of CD8^+^ T cells and M1 macrophages in the Immunity H group compared to the Immunity L group (Figure 1E). So, all these results showed that this bladder cancer unsupervised grouping performed well in distinguishing the difference of immune infiltration between samples and was suitable for further analysis.

### Identification of differentially expressed immune infiltration related lncRNAs

**Figure 2:**
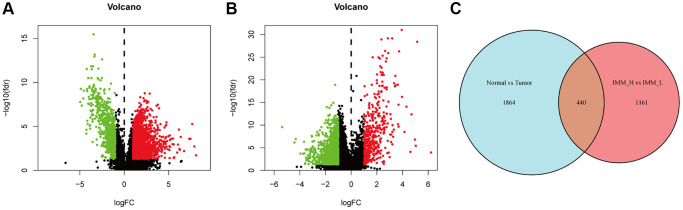
**Analysis of the differentially expressed lncRNAs.** (**A**) The volcano plot showed that 1699 and 635 genes were up-regulated and down-regulated between bladder cancer and paracancerous tissues. Each red dot showed an up-regulated gene and green showed downregulated genes (fold change >2, *p* < 0.05). (**B**) Consistent with Figure 3A, the volcano plot showed that 414 and 1187 genes were up-regulated and down-regulated between high and low immune cell infiltration group. (**C**) After taking an intersect, we obtained a total of 440 differentially expressed immune infiltration-related lncRNAs.

We firstly applied the criteria of |log_2_FC| > 1 and FDR <0.05 to screen the differentially expressed lncRNAs between bladder cancer samples (*n* = 411) and paracancerous samples (*n* = 19). Thus, we discovered 1669 lncRNAs up regulated and 635 lncRNAs down regulated (Figure 2A). Later, the same criteria were conducted between the Immunity H/L groups, and 1601 differentially expressed lncRNAs were discovered (Figure 2B). Finally, we took an intersect of them and identified a total of 440 differentially expressed immune-infiltration-related lncRNAs (Figure 2C).

### Construction and assessment of 12 immune-infiltration-related lncRNA prognostic signature for BC

**Figure 3:**
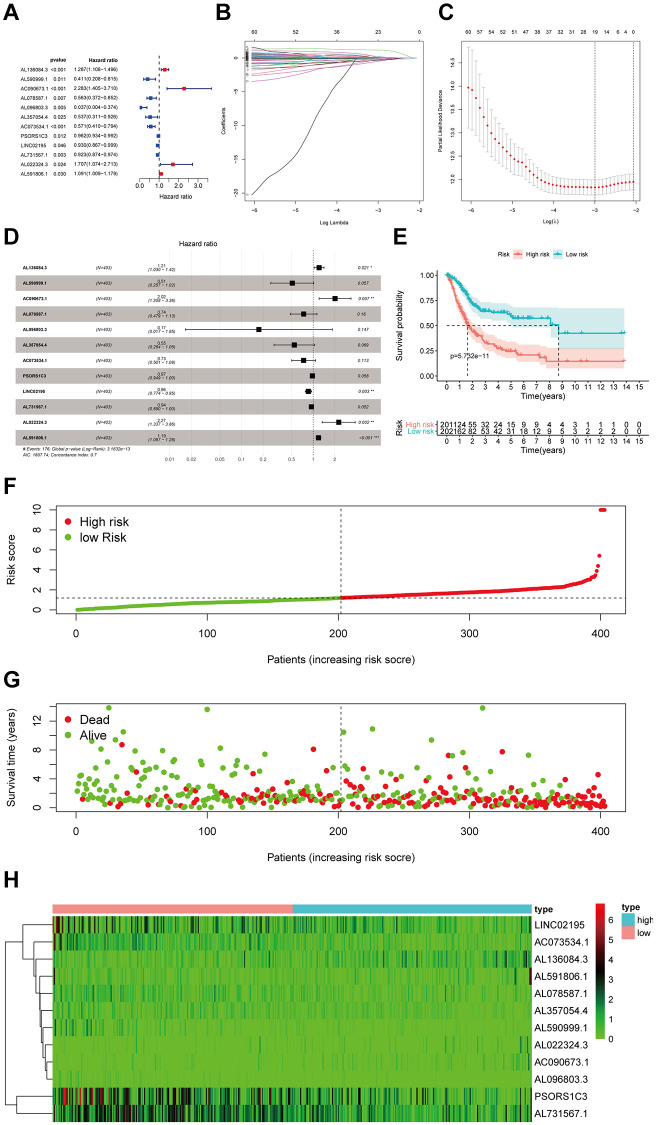
**Identification and assessment of immune-related lncRNA prognostic signature for bladder cancer.** (**A**) The HR and p-value of selected genes in the immune terms using the univariable Cox HR regression (Criteria: *p*-value <0.05). (**B**) The LASSO Cox analysis identified 19 lncRNAs most related to prognostics. (**C**) The 10-round cross-validation determined the optimal values of the penalty parameter. (**D**) The HR and *p*-value from the multivariable Cox HR regression prognostic signature. (**E**) Patients showed poor overall survival (OS) in the high-risk group (red) than those in the low-risk group (blue). (**F**) The risk curve of each sample reordered by risk score. (**G**) A sample survival overview using the scatter plot. The green dots represent survival and red represent death, respectively. (**H**) Heatmap showed the expression of the signature in the high-risk groups and low-risk groups. The pink and blue bars represented the low-risk group and the high-risk group. And the evolution from green to red represented the 0 to 6 level of gene expression.

We performed univariate Cox regression analysis of the 440 differentially expressed immune infiltration related lncRNAs. (Figure 3A). And 68 of them were found by applying the criterion of *p* < 0.05 (Original data sheet is in the Supplementary Table 1). Following this, we performed the least absolute shrinkage and selection operation (LASSO) regression to prevent overfitting and check 19 lncRNAs as appropriate variables (Figure 3B and 3C). Subsequently, we performed multivariate Cox regression to these 19 lncRNAs and developed a twelve-differentially expressed immune-infiltration-related lncRNAs signature (Figure 3D). The detailed information of these 12 lncRNAs was shown in Table 1 and the univariate regression results of these 12 lncRNAs were shown in Figure 3A. In addition, the risk score of each sample was calculated by the following formula: Risk score = 0.19 × *AL*136084.3 − 0.67 × *AL*590999.1 + 0.70 × *AC*090673.1 − 0.31 × *AL*078587.1 − 1.74 × *AL*096803.3 − 0.61 × *AL*357054.4 − 0.31 × *AC*073534.1 − 0.03 × *PSORS*1*C*3 − 0.15 × *LINC*02195 − 0.06 × *AL*731567.1 + 0.82 × *AL*022324.3 + 0.17 × *AL*591806.1 (Table 1). High-/low-risk groups were determined in samples according to the median risk-score. Kaplan-Meier curve showed that the overall survival (OS) of patients with high-risk is much lower than patients with low-risk, suggesting ab effective prognostic characteristics of risk score (*p* = 5.732e−11) (Figure 3E). The risk score distribution and corresponding scatterplot were together to show each bladder cancer sample’s risk and survival status. And we could see a higher risk score and mortality rate in high-risk groups (Figure 3F and 3G). Also, the heatmap suggested that there existed different expression between high-/low-risk groups (Figure 3H). In total, all these results confirm that this 12 immune-infiltration-related lncRNAs signature performs well in the prognosis prediction.

**Table 1:** The detailed information of the 12 immune infiltration-related lncRNAs used to construct the prognostic signature.

**Gene symbol**	**Ensemble ID**	**Gene_biotype**	**Coef**
***AL136084.3***	ENSG00000270412	antisense (lncRNA)	0.189106707332803
***AL590999.1***	ENSG00000235033	antisense (lncRNA)	−0.670726398604066
***AC090673.1***	ENSG00000197301	antisense (lncRNA)	0.700772620519985
***AL078587.1***	ENSG00000231081	lincRNA	−0.307233454792126
***AL096803.3***	ENSG00000273198	lincRNA	−1.74365287679752
***AL357054.4***	ENSG00000272463	lincRNA	−0.605876987647513
***AC073534.1***	ENSG00000276030	lincRNA	−0.309034938121597
***PSORS1C3***	ENSG00000204528	sense_intronic	−0.025963967640208
***LINC02195***	ENSG00000236481	lincRNA	−0.154404853480445
***AL731567.1***	ENSG00000231964	antisense (lncRNA)	−0.0581907270818915
***AL022324.3***	ENSG00000272942	lincRNA	0.820130537909633
***AL591806.1***	ENSG00000228917	antisense (lncRNA)	0.170471414924474

Notes: Antisense: Transcripts that overlap the genomic span (i.e., exon or introns) of a protein-coding locus on the opposite strand. Sense intronic: A long non-coding transcript in introns of a coding gene that does not overlap any exons. lincRNA (long intergenic ncRNA): Transcripts that are long intergenic non-coding RNA locus with a length >200 bp. Requires lack of coding potential and may not be conserved between species.

### 12 immune-infiltration-related lncRNAs signature can be an independent prognostic factor in BC

**Figure 4:**
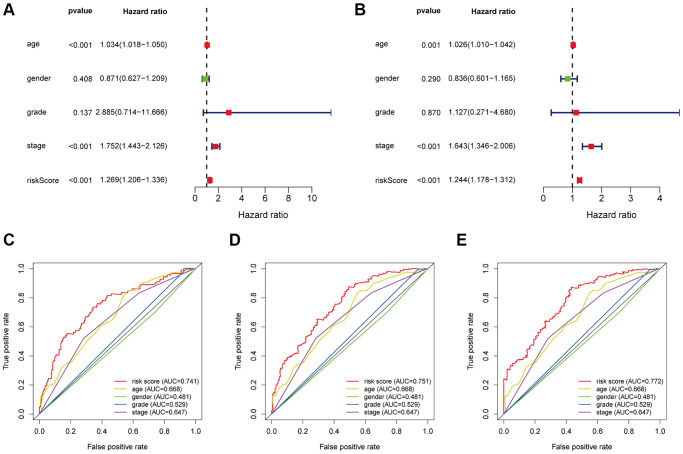
**Evaluate the independent prognostic value of the risk score by using the Cox regression analysis.** The (**A**) univariate cox regression and (**B**) multivariate cox regression analysis of age, gender, grade, and risk score. Calculate the AUC for age, gender, grade, and risk score of the total survival according to the multivariate time-dependent ROC curve for 1-year (**C**), 3-years (**D**) and 5-years (**E**).

Univariate and multivariate Cox regression were conducted to investigate if there was no association between 12 immune-related lncRNAs and clinicopathological factors or not. From the result, the HR (hazard ratio) and its 95% CI were 1.269 (1.206–1.336) in univariate Cox proportional regression (*p* < 0.001), and 1.244 (1.178–1.312) in multivariate Cox proportional regression (*p* < 0.001), separately, showing that this signature can perform as an independent prognostic element in bladder cancer patients (Figure 4A and 4B). Besides, time-dependent receiver operating characteristics (ROC) analysis was applied and plotted to compare the specificity and sensitivity of this signature with others clinical factors. The areas under the ROC curve (AUC) of the risk score in 1-. 3-, and 5-years were 0.741, 0.751 and 0.772, showing the 12 lncRNAs signature was more reliable than those commonly used clinicopathological factors including age, gender, grade, and stage (Figure 4C–4E). In total, both these two results suggested that the 12 lncRNAs signature could serve as an effective independent prognostic element for patients with bladder cancer.

### Relevance between 12 immune-infiltration-related lncRNAs signature and the immune cell infiltration

**Figure 5:**
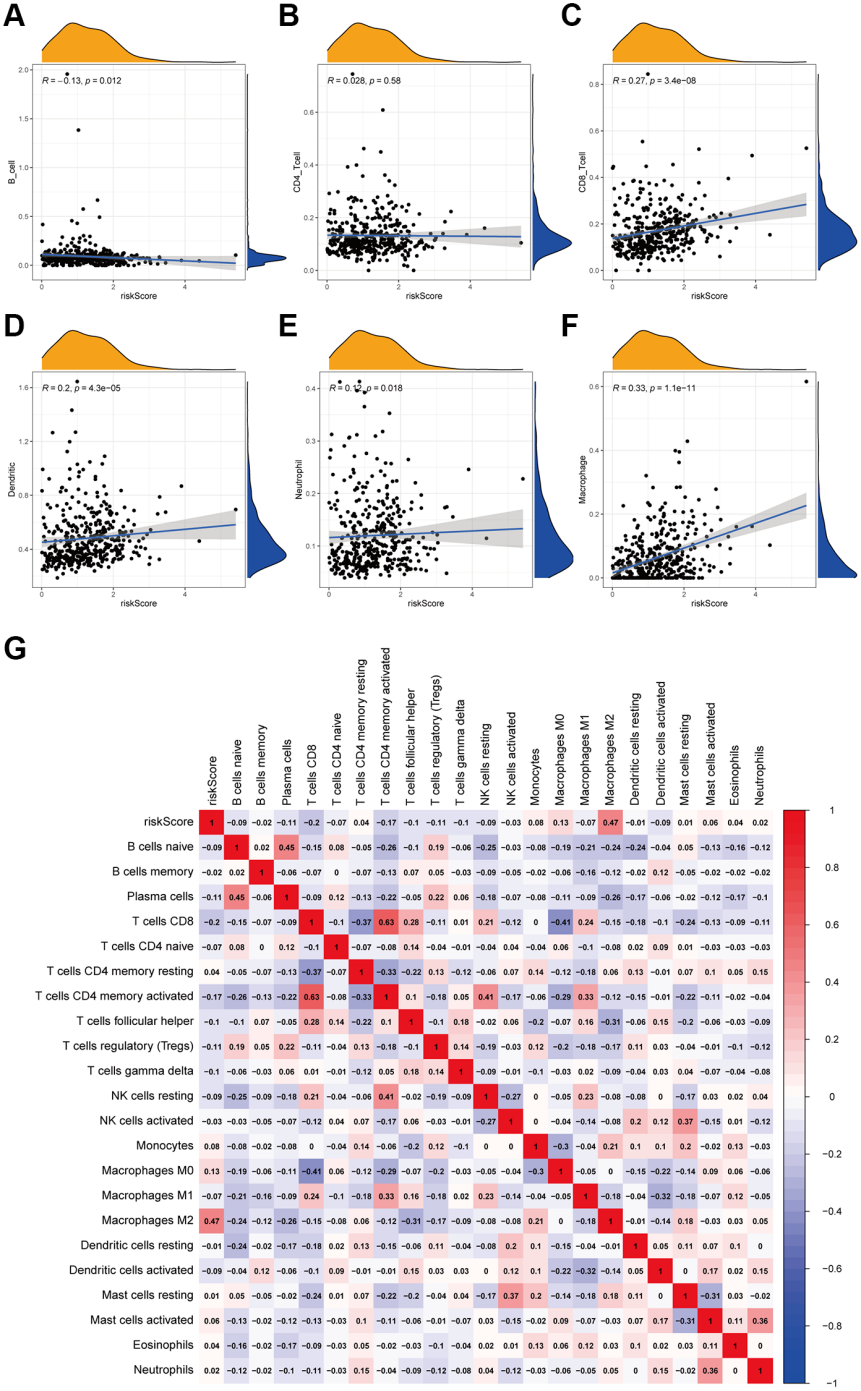
**Correlation between the 12 lncRNA prognostic signature for bladder cancer and the infiltration of immune cell subtypes.** The correlation values of all the immune cells with risk score. (**A**) B cells. (**B**) CD4+ T cell. (**C**) CD8+ T cell. (**D**) Dendritic. (**E**) Neutrophil. (**F**) Macrophage. (**G**) The correlation of immune cell infiltration and risk scores according to the infiltration results estimated by CIBESORT.

To investigate the correlation between this 12 immune-infiltration-related lncRNAs signature and the corresponding immune infiltration, we took another algorithm (TIMER) to assess the immune cell infiltration of each sample. Here in Figure 5A–5F, the infiltration of CD8+ T cells, dendritic cells and macrophages were significantly positive correlated with risk scores. Besides, we also used CIBESORT to analyzed the immune infiltration. And Figure 5G showed the more detailed relationship between different types of immune cells and risk scores, suggesting that the correlation co-efficient of macrophages M2 was also positive with risk scores, respectively, the same as the results showed in TIMER. In summary, these findings suggested that this 12-immune-infiltration related lncRNAs signature was associated with the immune infiltration.

### Nomogram and drug response

**Figure 6:**
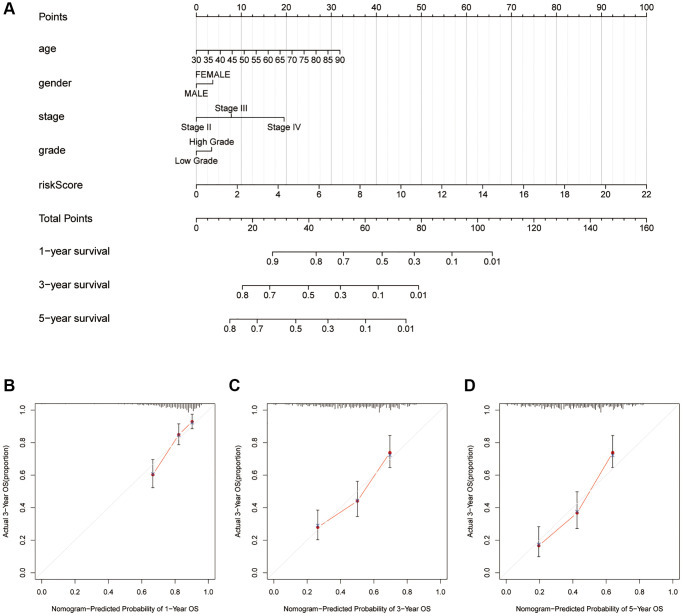
**Quantitively analyze the overall survival of patients with bladder cancer.** (**A**) Nomogram considering risk score and several common-used clinicopathological factors. Calibration curves for 1-yaer (**B**), 3-years (**C**), 5-years (**D**).

**Figure 7:**
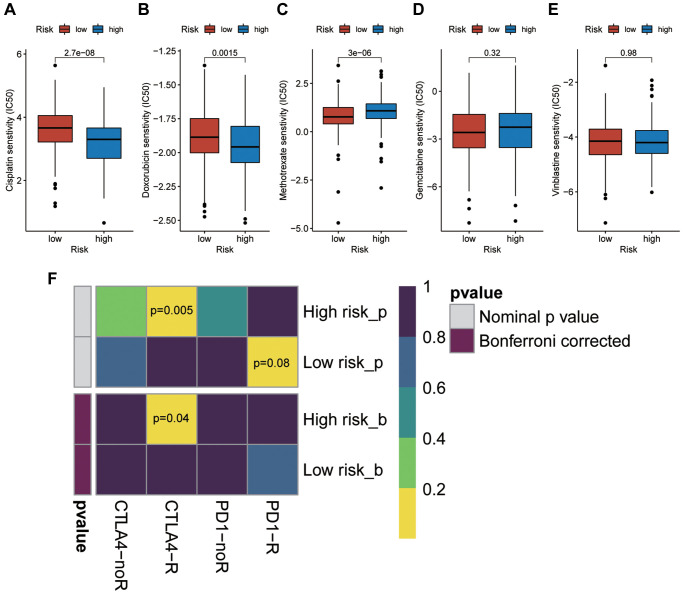
**Prediction of the sensitivity of high risk patients to chemotherapy drugs and immunotherapy.** (**A**) Cisplatin. (**B**) Doxorubicin. (**C**) Methotrexate. (**D**) Gemcitabine. (**E**) Vinblastine. (**F**) Immune checkpoint receptor: PD1-R, PD1-noR, CTLA4-R, ctal4-noR.

It seems the risk score is the most weighted factor in the nomogram (Figure 6A), and all of the calibration curves show a consistency between the survival predicted by this nomogram and the actual survival rates (Figure 6B–6D). Besides, we performed drug sensitivity prediction. As shown in the Figure 7A–7E, the ordinate represents IC50, so the smaller the IC50, the more sensitive it is to drugs. So, it is interesting so to see that though patients with high risk scores are associated with a poor prognosis, they showed a more sensitive response to the cisplatin and doxorubicin than patients with low risk scores (Figure 7A and 7B), while they are less sensitive to the methotrexate (Figure 7C). The response to both gemcitabine and vinblastine are no differences (Figure 7D and 7E). Similarly, in our immunotherapy response prediction, the high-risk group showed significantly better response to anti-CTLA4 treatment, while the low-risk patients were more sensitive to PD1 monoclonal antibody (Figure 7F).

## DISCUSSION

BC is one of the common urinary tumor accounts for high prevalence and recurrence rate [23]. BC tissue consists of cancer cells and other stromal cells and immune cells. Among them, immune cells have been proved functioned in tumor progression and prognosis [9]. Besides, TNM staging system cannot distinguish the survival and treatment response of patients accurately that it only considered the anatomical information and ignore the significant role of genetic background, [24]. Thus, scientists devoted to identifying and developing molecular biomarkers for the diagnosis and prognosis in cancer patients [25, 26]. There is significant meaning for discovering the mechanism of the progression in bladder cancer, and the treatment and prognosis of it. Our study focuses more on the immune infiltration related lncRNAs and their interaction with the immune cells. In this study, we identified and verified a 12 immune-infiltration-related lncRNAs as prognostic signature in patients with bladder cancer.

We firstly constructed an unsupervised bladder cancer grouping, dividing into high and low immune infiltration clusters. Then we discovered that there were significant differences in Tumor Purity, ESTIMATE Score, Immune Score, and Stromal Score between these two clusters. Interestingly, the expression of HLA and CD274, also the algorithm of CIBERSORT verified the reliability of this unsupervised immune cluster.

In recent years, under the deep studies in transcriptome sequencing, we have known that around 80% of the transcripts in human genome are noncoding genes, such as lncRNAs, miRNAs, circRNAs, and tsRNAs. Among them, lncRNAs were shown to be associated with the progression, prognosis of bladder cancer [19]. In this study, we identified 12 immune-infiltration-related lncRNAs associated with the prognosis of patients with bladder cancer. Among them, LINC02195 is reported as a favorable prognostic marker in head and neck squamous cell carcinoma [27]. Also, lncRNA PSORS1C3 is discovered expressed regulated by the expression of transcription factor OCT4 in non-pluripotent cells [28]. Having confirmed the significant role of these immune-infiltration related lncRNAs in several biological processes, we constructed the prognostic signature and verified the efficacy of this signature by the univariate and multivariate Cox analysis. Notably, time dependent ROC curves (for 1, 3, 5 years) were plotted to compare the prognosis efficacy between this signature with other common-used clinical factors of patients with bladder cancer, including age, gender, pathological grade and stage.

Several studies suggested that tumor-infiltrating lymphocytes are associated with tumor recurrence, progression, and drug response [29, 30]. And it was found that tumor-infiltrating immune cells hold a high infiltration proportion in several types of cancer, for example, breast cancer and skin melanoma [20, 31]. Moreover, the immune infiltration are the main targets of the immunotherapy [32]. In this study, we found that the B cells were significantly negatively-correlated with risk score, and M2 macrophages was positively. B cells, regarded as effector cells of anti-tumor cellular immunity, low infiltration in tumor tissue caused a poor prognosis. On the other hand, M2 macrophages could enhance cell growth [33]. Therefore, these results revealed two potential mechanisms causing worse prognosis in high-risk patients and indicated the potential therapeutic targets in patients with bladder cancer.

In addition, we analyzed the sensitivity of high-risk patients to chemotherapeutic drugs. Interestingly, we found that high risk patients are highly sensitive to cisplatin and doxorubicin. Similarly, these patients were also more sensitive to anti-CTLA4 immunotherapy. We speculate that this may be related to different immune cell infiltration and immune checkpoint expression between high and low risk groups. Therefore, although high risk patients showed a poorer prognosis, these results could provide us with new ideas for targeted treatment among them.

Inevitably, there are some limitations in our research that should be pointed out. Firstly, the 12-lncRNA prognostic signature was only obtained and validated in the TCGA dataset. Secondly, more patient datasets are supposed to verify the performance of the 12-lncRNA prognostic signature. Besides, all the findings need to be verified by more analysis in order to increase authenticity.

In conclusion, our study identified a novel twelve-immune infiltration-related lncRNA signature for bladder cancer. We also found that different score-based groups showed different immune infiltration. These findings may reveal a potential target for the prognostic evaluation of patients with bladder cancer and provide more ideas for further studies on tumor immunity in bladder cancer.

## MATERIALS AND METHODS

### Data sources

TCGA_BLCA dataset were retrieved and downloaded in the fragments per kilobase of per million format (FPKM) from the TCGA database (https://portal.gdc.cancer.gov/), the corresponding clinical information containing age, gender, survival status, overall survival, pT stage, pN stage, pM stage, and AJCC stage were also downloaded from TCGA database. A total of 403 bladder cancer patients were enrolled in this research (Table 2). Notably, we obtained annotation gene sets consisting of 29 immune related gene sets considering both immune cells and immune related pathways or functions. Then we performed ssGSEA analysis to emphasize the integrative immune cells, immune related pathways, and immune related functions of each bladder cancer samples by the R package “GSVA”. Following this, all the bladder cancer samples were unsupervised clustered and divided into two clusters defined as high/low immune infiltration group according to the ssGSEA results.

**Table 2:** Clinical characteristics of the BLCA patients.

	**Overall**	**High risk**	**Low risk**	***p***
*N*	403	201	202	
Age (mean (SD))	68.06 (10.60)	69.54 (10.05)	66.58 (10.94)	0.005
Gender = Female/Male (%)	105/298 (26.1/73.9)	60/141 (29.9/70.1)	45/157 (22.3/77.7)	0.106
Grade (%)				<0.001
High Grade	380 (94.3)	197 (98.0)	183 (90.6)	
Low Grade	20 (5.0)	1 (0.5)	19 (9.4)	
Unknown	3 (0.7)	3 (1.5)	0 (0.0)	
Stage (%)				<0.001
Stage I	1 (0.2)	0 (0.0)	1 (0.5)	
Stage II	127 (31.5)	44 (21.9)	83 (41.1)	
Stage III	138 (34.2)	74 (36.8)	64 (31.7)	
Stage IV	133 (33.0)	82 (40.8)	51 (25.2)	
Unknown	4 (1.0)	1 (0.5)	3 (1.5)	
T (%)				0.001
T0	1 (0.2)	0 (0.0)	1 (0.5)	
T1	3 (0.7)	1 (0.5)	2 (1.0)	
T2	37 (9.2)	8 (4.0)	29 (14.4)	
T2a	25 (6.2)	9 (4.5)	16 (7.9)	
T2b	56 (13.9)	27 (13.4)	29 (14.4)	
T3	42 (10.4)	23 (11.4)	19 (9.4)	
T3a	69 (17.1)	32 (15.9)	37 (18.3)	
T3b	80 (19.9)	49 (24.4)	31 (15.3)	
T4	10 (2.5)	9 (4.5)	1 (0.5)	
T4a	43 (10.7)	27 (13.4)	16 (7.9)	
T4b	5 (1.2)	4 (2.0)	1 (0.5)	
TX	1 (0.2)	0 (0.0)	1 (0.5)	
Unknown	31 (7.7)	12 (6.0)	19 (9.4)	
M (%)				0.015
M0	193 (47.9)	82 (40.8)	111 (55.0)	
M1	11 (2.7)	8 (4.0)	3 (1.5)	
MX	197 (48.9)	110 (54.7)	87 (43.1)	
Unknown	2 (0.5)	1 (0.5)	1 (0.5)	
N (%)				0.028
N0	234 (58.1)	104 (51.7)	130 (64.4)	
N1	46 (11.4)	31 (15.4)	15 (7.4)	
N2	75 (18.6)	43 (21.4)	32 (15.8)	
N3	7 (1.7)	5 (2.5)	2 (1.0)	
NX	36 (8.9)	15 (7.5)	21 (10.4)	
Unknown	5 (1.2)	3 (1.5)	2 (1.0)	
RiskScore (median [IQR])	1.18 [0.72, 1.76]	1.76 [1.50, 2.14]	0.72 [0.43, 0.95]	<0.001

### Characteristics of the immune grouping

Having obtained the immune grouping of each sample, we would like to verify the effective ness of this immune cluster. Thus, we firstly conducted ESTIMATE analysis to emphasize the corresponding immune score, stromal score, estimate score, and tumor purity of each sample according to their transcriptional expression. Then, all these immune related scores were compared between these two immune groups. Besides, the gene expression of immune checkpoint including human leukocyte antigen (HLA) and CD274 (PD-L1) were compared between high immune cell infiltration group (Immunity H) and low immune cell infiltration group (Immunity L) to further verifying the effectiveness of the immune infiltration groups. Moreover, CIBERSORT algorithm was performed to estimate the detailed immune cell infiltration status of each sample, and Wilcoxon test was carried out to investigate the differential immune cell infiltration between these two immune infiltration groups.

### Identification of differentially expressed immune-infiltration-related lncRNAs in BC

According to the immune infiltration groups clustered by the ssGSEA results, as mentioned above, the lncRNA expression profile data extracted and annotated from the transcriptome file were ranked from the Immunity H group to the Immunity L group. Then the immune infiltration related lncRNA were identified by the differential expression analysis between Immunity H group and Immunity L group. Besides, differentially expressed lncRNAs were identified by the same methodology between tumor tissue and normal adjacent tissue. Notably, both the filter criteria were the |log_2_FC| >1 and FDR < 0.05. Following this, we took an intersection of these immune infiltration related lncRNAs and differentially expressed lncRNAs to obtain the final differentially expressed immune infiltration related lncRNAs for further analysis.

### Further identification of immune infiltration-related lncRNA prognostic signature in BC

Having obtained the differentially expressed immune infiltration related lncRNAs, we merged the expression value with the detailed survival information of each sample. Then, we conducted univariate cox regression in order to screen if those differentially expressed lncRNAs have prognostic value or not. Besides, to avoid over-fitting, we subsequently carried the Least Absolute Shrinkage and Selection Operator (LASSO) regression to obtain the appropriated variables for further signature construction. Finally, multivariate cox regression was performed to construct the immune infiltration related lncRNA prognostic signature. A corresponding risk score formula was also established as follow:


riskScore=∑i=1ncoef(i) ⋅ exp(i)

Then each patient received a risk score according to this formula. The median value of all patients were set as the threshold, and all patients were divided into the high or low risk score group that higher than the median value is high risk and the lower represents low risk. Kaplan-Meier survival curves were plot and log-rank test was used to check whether this risk stratification is associated with the overall survival. Besides, univariate and multivariate cox regression were carried out to investigate whether this risk score could serve as an independent prognostic factor.

### Correlation between prognostic signature and detailed immune infiltration

Here we took two differential methods to estimate the immune cell infiltration of each sample, and they were TIMER and CIBERORT. Among them, the immune cells calculated by TIMER was downloaded from the TIMER database (https://cistrome.shinyapps.io/timer/). The CIBERSORT results were calculated by the CIBERSORT algorithm. Following these two methods, PEARSON correlation test was carried out to further investigating the correlation between risk score and these detailed immune cells infiltration.

### Nomogram and drug response

To emphasize the overall survival of patients with bladder cancer more quantitatively, we re-checked the corresponding clinical data of each patient and assembled a nomogram considering risk score and several common-used clinicopathological factors including age, gender, stage, and grade. Calibration curves for 1-, 3-, 5-years were also plotted to examine the accuracy of the nomogram. Finally, we separately predicted the drug response to chemotherapy by R package “ProPhetic” and the drug response to immunotherapy by submap algorithm [34]. Then compared the drug response to both chemotherapy and immunotherapy between high and low risk patients.

### Statistical analysis

All statistical analysis was applied by R program version 4.0.2. Mean ± standard deviation was applied to describe the distribution of the continuous variables following normal distribution while the median (range) was used for continuous variables following abnormal distribution. Counts and percentages were used to describe the distribution of categorical variables. Two-tailed *p* < 0.05 was considered with statistical significance.

### Data availability

Source data of this study were derived from the public repositories, as indicated in the section of “Materials and Methods” of the manuscript. And all data that support the findings of this study are available from the corresponding author upon reasonable request.

## Supplementary Materials

Supplementary Table 1
