# Supplementary material for: Identification and validation of a twelve immune infiltration-related lncRNA prognostic signature for bladder cancer
Source: Aging (Albany NY). 2022 Feb 14;14(3):1492–507. doi: 10.18632/aging.203889 (PMC8876923; doi:10.18632/aging.203889)
Supplement: Supplementary Table 1 [file aging-14-203889-s001.pdf]

SUPPLEMENTARY TABLE

Supplementary Table 1. Original data sheet for result of univariate cox regression analysis.

HR.95L	HR.95H	*p* value
0.037373	0.863567	0.032107
0.071252	0.751761	0.014905
0.823655	0.965727	0.004817
1.107931	1.495544	0.00097
0.778513	0.961014	0.006931
0.20777	0.814923	0.010866
0.041163	0.985738	0.047963
1.405309	3.709919	0.000856
4.718543	58.36037	1.20E-05
0.383831	0.873397	0.009178
1.00403	1.062617	0.025223
0.001346	0.988583	0.049208
1.08E-24	0.092859	0.032647
0.121546	0.934556	0.036615
1.00434	1.171122	0.03841
0.025316	0.934202	0.041961
0.096149	0.761124	0.013244
0.383026	0.996425	0.048308
0.065267	0.859976	0.028575
0.267092	0.877746	0.016867
1.60947	8.1431	0.001867
0.720106	0.956215	0.009913
0.955247	0.997075	0.025889
0.741827	0.993449	0.040543
0.345136	0.977203	0.040676
0.372394	0.852072	0.006566
1.045542	1.486055	0.01403
1.08179	2.422022	0.019164
0.016615	0.740481	0.023199
0.050064	0.829233	0.026321
0.000711	0.886163	0.042722
0.136897	0.55985	0.000351
0.00369	0.374232	0.005205
0.725512	0.927459	0.001567
3.36E-06	0.63273	0.035057
0.046447	0.569637	0.004511
0.417327	0.976494	0.038486
0.396445	0.887949	0.011159
0.348229	0.890129	0.014441
0.311026	0.925908	0.025314
0.47903	0.886868	0.006449

0.377071	0.943865	0.02733
0.410005	0.794244	0.000882
0.558959	0.929608	0.011654
1.031049	1.13786	0.001494
0.305928	0.981087	0.04295
0.019117	0.670647	0.016388
0.950755	0.996051	0.021835
0.933923	0.991654	0.012153
0.689591	0.939781	0.006024
0.51755	0.899356	0.006679
1.020366	1.116804	0.004582
0.070603	0.707854	0.010849
0.118042	0.902779	0.031003
0.866871	0.998727	0.046019
1.038628	1.40632	0.014279
0.118134	0.855993	0.023345
0.874245	0.973553	0.00332
1.074067	2.713456	0.023686
1.009407	1.052308	0.004487
0.528319	0.915661	0.009654
1.008668	1.179039	0.029501
0.867436	0.968161	0.001842
0.394578	0.881566	0.010034
0.859295	0.997386	0.042478
1.037484	1.573768	0.021102
0.138538	0.905688	0.030252
1.666265	5.708041	0.000336
